# Application of Fuzzy Logic-Based Expert Advisory Systems in Optimizing the Decision-Making Process for Material Selection in Additive Manufacturing

**DOI:** 10.3390/ma18020324

**Published:** 2025-01-13

**Authors:** Kinga Skrzek, Emilia Mazgajczyk, Bogdan Dybała

**Affiliations:** Centre for Advanced Manufacturing Technologies (CAMT/FPC), Faculty of Mechanical Engineering, Wrocław University of Science and Technology, Łukasiewicza 5 St., 50-370 Wroclaw, Poland; emilia.mazgajczyk@pwr.edu.pl (E.M.); bogdan.dybala@pwr.edu.pl (B.D.)

**Keywords:** fuzzy logic, expert advisory systems, intelligent manufacturing, additive manufacturing, 3D printing, decision-making optimization, Industry 4.0

## Abstract

In the era of Industry 4.0, additive manufacturing (AM) technology plays a crucial role in optimizing production processes, especially for small- and medium-sized enterprises (SMEs) striving to enhance competitiveness. Selecting the appropriate material for AM is a complex process that requires considering numerous technical, economic, and environmental criteria. Fuzzy logic-based advisory systems can effectively support decision-making in conditions of uncertainty and subjective user preferences. This study presents a developed advisory system model that uses the Analytic Hierarchy Process (AHP) method and triangular and trapezoidal membership functions, enabling dynamic adjustment of criterion weights. The results demonstrated that the system achieved 85% alignment with user preferences, confirming its effectiveness. Future research may focus on integrating fuzzy logic with machine learning algorithms to further enhance the system’s precision and flexibility.

## 1. Introduction

Additive manufacturing (AM) has emerged as a transformative technology aligned with the goals of Industry 4.0, offering customized production, reduced waste, and enhanced efficiency. For small- and medium-sized enterprises (SMEs), AM presents opportunities to innovate and compete through scalable and cost-effective production methods. However, adopting AM technologies introduces significant challenges, particularly in material selection, which is a critical factor influencing product quality, cost efficiency, and environmental impact. The material selection process is inherently complex, as it requires balancing multiple criteria such as mechanical properties, cost, and sustainability, while operating under resource constraints typical of SMEs [1,2].

SMEs face several unique challenges in this context. Unlike large enterprises, SMEs often operate with limited access to comprehensive datasets on material properties and market availability. Inconsistent or incomplete data related to tensile strength, thermal stability, and cost variability complicate decision-making. Additionally, the selection process is frequently influenced by subjective user preferences and organizational priorities, which can lead to bias and suboptimal choices. For example, decision-makers may prioritize short-term cost savings over long-term performance due to budget constraints, which may not align with optimal material performance goals [3].

Traditional decision-making methods, such as weighted scoring or simple multi-criteria decision-making (MCDM) approaches like the Analytic Hierarchy Process (AHP) or Simple Multi-Attribute Rating Technique (SMART) often fall short in addressing these challenges. These methods typically assume precise, deterministic data and fixed weightings for criteria, making them less effective in the face of data uncertainty and dynamic preferences [4]. Furthermore, they lack the adaptability to adjust to changing project requirements or to incorporate subjective user preferences effectively. For instance, while AHP provides a detailed and consistent framework for assigning weights through pairwise comparisons, it can become cumbersome when dealing with uncertain data [5]. In contrast, SMART is more efficient for simpler scenarios but is more susceptible to user bias due to direct weight assignments [6,7].

To overcome these limitations, recent advancements have focused on hybrid decision-support systems that integrate fuzzy logic with artificial intelligence (AI) techniques. These systems excel in handling uncertainty and accommodating subjective inputs by providing flexible, rule-based reasoning [8]. Over the past few years, research has demonstrated the effectiveness of combining fuzzy logic with machine learning (ML) algorithms, such as neuro-fuzzy systems and genetic-fuzzy hybrids [9]. These hybrid systems can dynamically adjust membership functions and criteria weights based on historical data and real-time user feedback, improving decision accuracy and relevance for SMEs [10,11]. For example, reinforcement learning (RL) integrated with fuzzy systems enables continuous adaptation to evolving market conditions, which is particularly beneficial for long-term material selection strategies [12].

In this context, fuzzy logic-based systems offer a significant advantage by capturing the uncertainty and variability inherent in the material selection process. Unlike traditional methods that require crisp, exact data, fuzzy logic uses membership functions—such as triangular and trapezoidal functions—to express degrees of truth or satisfaction [13]. These membership functions allow decision-makers to model subjective preferences more accurately and to evaluate material options even when data are imprecise or incomplete [14].

This research builds on these advancements by presenting an expert advisory system that combines fuzzy logic and AHP to address SMEs’ specific challenges in AM material selection. The system leverages the strengths of AHP’s hierarchical decomposition and consistency checks while benefiting from fuzzy logic’s ability to handle uncertainty [15,16]. Triangular and trapezoidal membership functions are employed to dynamically model user preferences and criterion weights. Through this approach, the system achieves improved alignment with user needs and offers actionable insights that traditional methods cannot provide [17].

Furthermore, by incorporating both objective technical parameters and subjective user preferences, the system provides a more holistic and adaptable decision-making framework. This adaptability ensures that SMEs can make informed material choices that balance cost, performance, and sustainability, ultimately enhancing their competitiveness in the rapidly evolving AM landscape [18].

## 2. Materials and Methods

This section describes the research process and methods used to develop an expert advisory system that supports material selection decisions in additive manufacturing for SMEs. The fuzzy logic foundation of the system allows for flexible modeling of user preferences while accommodating uncertain or incomplete input data [19].

In the developed expert advisory system, three distinct types of criteria are identified: MINSIMP, MAXSIMP, and MAXINV. The first of these is the MINSIMP criterion type [20,21]. For this type of criterion, the optimal value is the smallest possible, and the most critical variations in the degree of criterion fulfillment occur near the minimum point of the criterion’s domain [22]. The domain of the MINSIMP criterion is divided into characteristic points, which are calculated using a specified formula. These points help assess how well a particular solution meets the criterion by focusing on the proximity to the minimum value, reflecting the importance of minimizing this criterion in the overall decision-making process [23]. This approach ensures that the system prioritizes solutions that achieve the lowest possible value for this specific type of criterion, while still allowing for the flexible evaluation of options through fuzzy logic:

(1) $$P_{v}=P_{min}+\left(P_{max}-P_{min}\right)\times \frac{2^{v}}{64},v=0,1,\ldots ,6$$

The characteristic points in the domain of the MINSIMP criterion are calculated using the following relationship, where

- P_v_ is the argument value of the characteristic point;
- P_min_ is the minimum value of the criterion domain;
- P_max_ is the maximum value of the criterion domain.

These points are distributed to assess how close a particular solution is to the minimum value, ensuring that the most important differences in the degree of criterion fulfillment are located around this minimum [24]. By calculating these characteristic points, the system can model the criterion in a way that reflects its significance in the decision-making process.

The degree of criterion fulfillment for the MINSIMP criterion type is calculated using the following formula:

g = 10 − v,(2)

where

- g is the degree of criterion fulfillment, on a scale from 4 to 10;
- v is the normalized value of the criterion being assessed.

Thanks to the implementation of the formula above, the degree of criterion fulfillment is expressed on a scale frequently used by decision-makers, providing a familiar and intuitive framework for evaluating criteria.

The second type of criterion introduced by the SMART method is the MAXSIMP criterion type. For this criterion, the optimal value is the highest possible, and the most significant differences in the degree of criterion fulfillment occur near the maximum point of the criterion’s domain [25].

The characteristic points in the domain of the MAXSIMP criterion are calculated using the following formula:

(3) $$P_{v}=P_{max}-\left(P_{max}-P_{min}\right)\times \frac{2^{v}}{64},v=0,1,\ldots ,6$$

As with the MINSIMP criterion, the degree of fulfillment for the MAXSIMP criterion is calculated using Formula (3), but in this case, it prioritizes solutions with values closer to the maximum, reflecting the system’s preference for maximizing the criterion.

This type of criterion is used to describe a condition where the optimal value is as large as possible, but the most important differences in the degree of criterion fulfillment occur near the minimum point of the criterion domain [26]. The characteristic points of this criterion domain are calculated similarly to the MINSIMP criterion type. However, the degree of criterion fulfillment is calculated based on the formula:

g = 4 + v,(4)

The values for the degree of criterion fulfillment range from 4 to 10. To convert these into normal fuzzy sets, a normalization process is required [27,28]. This process is performed by dividing the calculated values by 10, ensuring that the results fit within the range of 0 to 1, which is typical for fuzzy sets.

The third type of the criterion is MAXINV criterion type. This type of criterion is used to describe a condition in which the optimal value is as big as possible and the most important differences in the degree of criterion fulfillment are around the minimum point of the criterion domain. 

In the expert advisory system, the calculation of the total grade for a given variant involves the application of fuzzy set theory and defuzzification techniques. The fuzzy set FS is defined by its membership function μ(FS) and its domain of support x. The total grade of the analyzed variant is determined by employing the height operator, represented by the following Formula (5):

(5) $$G_{total}=\frac{\sum_{i=1}^{n}\mu \left({FS}_{i}\right)\cdot x_{i}}{\sum_{i=z}^{n}\mu \left({FS}_{i}\right),}$$

where

- G_total_ is the total grade for the analyzed variant;
- $\mu \left({FS}_{i}\right)$ is the membership function value of the i-th fuzzy set (degree of criterion fulfillment);
- x_i_ is the corresponding domain of support value for the i-th criterion;
- n is the total number of criteria.

**Figure 1:**
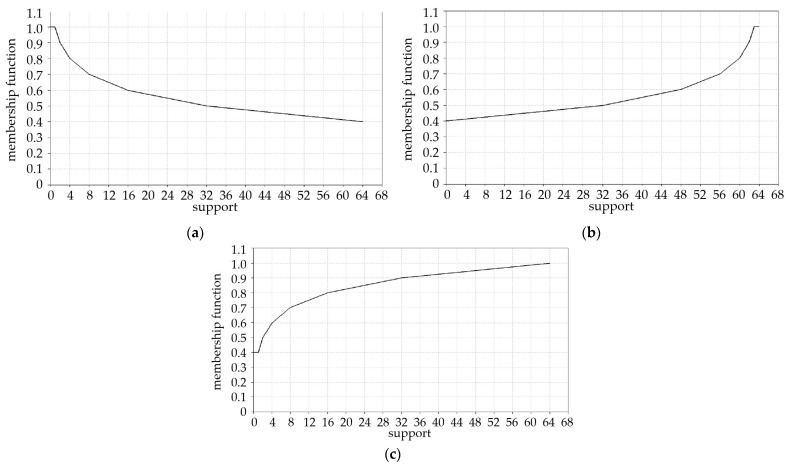
Fuzzy set for criterion where the optimal value is (**a**) the smallest one—MINSIMP; (**b**) the biggest one—MAXSIMP; (**c**) as big as possible—MAXINV [27].

The obtained values for the criteria fulfillment degree, calculated on the basis of Equations (2) and (4), range from 4 to 10. The shapes of the membership functions for each criterion type, reflecting the variations in the degree of fulfillment, are shown in Figure 1. These visual representations help in understanding how each criterion contributes to the overall decision-making process based on the different types of criteria: (a) MINSIMP, (b) MAXSIMP, and (c) MAXINV.

In the accumulation process, the T-norm operator is applied to combine the fuzzy sets obtained from n criteria. Then, the height operator merges these fuzzy sets to obtain a final crisp grade for the analyzed variant. This crisp grade is a numeric value representing the overall performance of the variant based on all the considered criteria [29,30].

The objective function of the expert advisory system incorporates all formulated criteria into the calculations. The optimization algorithm aims to maximize the total crisp grade of the selected technology variant.

The crisp grade of the variant can range from 4 to 10, and the results are interpreted linguistically, as shown in Table 1 [29].

The objective function in the expert advisory system aims to maximize the total crisp grade of the selected technology, incorporating all defined criteria. The resulting score allows for the classification of results on a qualitative scale from “Very Low” (VL) to “Very High” (VH). This evaluation system enables easier interpretation of results and supports the decision-making process by clearly indicating the quality level of the technology.

In summary, AHP is preferable for complex decision-making processes requiring detailed accuracy and consistency checks, such as the material selection process in additive manufacturing. In contrast, SMART is ideal for less complex decisions where speed is a priority. The decision to use AHP in this study was based on the need for precision in evaluating complex criteria for material selection. These criteria were evaluated and weighted using the AHP Formula (6) [30]:

(6) $$w_{i}=\frac{\sum_{j-i}^{n}\frac{A_{ij}}{\sum_{k=1}^{n}A_{kj}}}{n}$$

where

- w_i_ represents the weight of the i-th criterion;
- A_ij_ is the value in the comparison matrix for the i-th and j-th criteria;
- n is the total number of criteria.

This method ensures that weights are adjusted precisely, reflecting both the criterion’s significance and the expert evaluations (Table 2). The dynamically adjustable weights allow for the system’s adaptability to changing preferences or priorities within SMEs.

The advisory system is based on fuzzy logic, which enables the representation of imprecise and subjective information through membership functions. Each criterion was assigned a triangular or trapezoidal membership function, with boundary points defined based on historical data analysis and expert preferences. An example of a triangular membership function is shown below Formula (7) [31]:

(7) $$\mu \left(x\right)=\left\{\begin{array}{c} 1\\ \frac{x-a}{b-a}\\ \frac{c-x}{c-b}\\ 0 \end{array}\right.\;\begin{array}{c} for\;x\leq a\\ \begin{array}{c} for\;a<x\leq b\\ for\;b<x\leq c \end{array}\\ for\;x\geq c \end{array},$$

where

- a, b, c are the boundary points of the membership function for the selected criterion.

The advisory system relies on a set of “if… then…” decision rules, which allow for a flexible evaluation of the criteria values and the generation of recommendations. An example of a decision rule for material selection in additive manufacturing is as follows: if the material strength is high and production cost is low, then the preferred material is Material A.

An essential part of the study was the sensitivity analysis, which assessed the impact of changes in criterion weights on the final recommendations. The results indicate that the system remains stable with minor weight adjustments, which enhances its resistance to slight differences in criterion assessments.

The developed advisory system based on fuzzy logic employs triangular and trapezoidal membership functions due to their flexibility and computational efficiency. Triangular membership functions were selected for criteria where there is a clearly defined optimal point, with a linear increase and decrease in values on either side of this point. These functions are particularly suitable for criteria with well-defined minimum, maximum, and optimal values, such as production costs or material strength.

In contrast, trapezoidal membership functions were applied in cases where the optimal range of criterion values is broader and not confined to a single point. These functions allow for more flexible modeling of user preferences, especially when multiple values are acceptable, such as thermal stability or chemical resistance.

The boundary points for the membership functions were established based on two primary sources of information. The first source was an analysis of historical data from previous implementations of additive manufacturing (AM) technologies in SMEs. This analysis provided realistic ranges for key technical parameters.

The second source consisted of expert opinions from specialists in 3D printing and AM technologies. Interviews and workshops were conducted to ensure that the defined boundary points reflected current industry standards and practical requirements.

To validate the boundary points, simulation tests were performed, where users evaluated the system’s recommendations in simulated decision-making scenarios. The results achieved an 85% alignment with actual expert decisions, confirming the validity of the selected boundary points and the effectiveness of the chosen membership functions.

The Analytic Hierarchy Process (AHP) was integrated with fuzzy logic to provide precise and dynamic weighting of criteria. The process began with pairwise comparisons for all criteria, resulting in a preference matrix reflecting the relative importance of each criterion.

To ensure the reliability of these comparisons, the Consistency Ratio (CR) was calculated using Formula (8):

(8) $$CR=\frac{CI}{RI}$$

where CI is the Consistency Index, calculated as (Formula (9)):

(9) $$CI=\frac{\lambda_{max}-n}{n-1}$$

Here, *λ*_max_ is the largest eigenvalue of the comparison matrix, and n is the number of criteria. The consistency ratio was deemed acceptable if CR < 0.1. If the ratio exceeded this threshold, the comparisons were re-evaluated and adjusted to improve consistency.

The weights derived from AHP were then dynamically adjusted using fuzzy logic, allowing the system to respond flexibly to changing input data and user preferences.

In the implementation of the SMART method, a detailed sensitivity analysis was conducted to evaluate the impact of different scoring approaches on the system’s final recommendations. The analysis involved the following steps:

- Weight variability—the weights of each criterion were adjusted by ±10% to test the system’s response to changes;
- Scoring approaches—both linear and non-linear scoring scales were applied to examine how different methods of assigning scores influenced outcomes;
- Stability assessment—the system’s recommendations were compared across different scenarios to assess stability and reliability.

The results indicated that the system remained stable and provided consistent recommendations, confirming its robustness for dynamic decision-making environments typical of SMEs.

## 3. Results

At the beginning of the project, an analytical study was conducted to identify the key criteria essential for assessing the implementation of additive manufacturing technologies. These criteria were determined based on the specific needs of the company and encompass both customer requirements and technical parameters. Each criterion type and its associated arguments were carefully defined to ensure comprehensive evaluation and alignment with project goals [32]. The full list of customer requirements is outlined in Table 3, while the corresponding technical parameters are detailed in Table 4.

After defining the criteria, a method was developed to calculate their arguments. The genetic representation of the proposed solution serves as input for these calculations. To address uncertainties and the approximate nature of the criteria, the values of individual parameters were modeled as fuzzy sets. Additionally, constraints such as required intervals between technology upgrades or adjustments were modeled using L-type fuzzy sets. All defined fuzzy sets were integrated into the system to compute the assessment criteria values for the expert advisory system [33].

The results of the study highlight the effectiveness of the fuzzy logic-based system in the material selection process for additive manufacturing (AM). Through expert and user input, the system achieved an 85% recommendation accuracy rate, underscoring a high degree of alignment with actual user preferences in terms of material characteristics, cost efficiency, and overall adaptability to specific project needs. This accuracy is particularly noteworthy for SMEs that rely on precision in material selection to manage limited budgets and ensure quality in production processes [34].

The fuzzy logic system’s ability to accommodate data variability and user subjectivity is reflected in the degree of membership values assigned to each key criterion (Table 5). High membership degrees for parameters such as material strength and environmental impact reflect a consistent preference for robust, sustainable materials in AM processes—elements that align closely with the practical needs of SMEs.

The high degree of membership for material strength (0.82) indicates that users prioritize durability and robustness, which are crucial for the longevity and performance of AM products. Additionally, environmental impact scored 0.80, indicating a growing emphasis on sustainability, reflecting a trend towards environmentally conscious manufacturing practices [35]. The production cost criterion, with an average membership degree of 0.75, confirms the need for cost-effective solutions within the constraints typical of SMEs.

These values highlight that while users prioritize material performance and environmental responsibility, they also consider scalability and durability, albeit with slightly lower emphasis. Thus, the system’s structure and fuzzy criteria successfully capture the nuanced balance between performance and cost, critical for SMEs operating in competitive AM markets.

To assess the system’s performance comprehensively, results were compared with those obtained through traditional weighted scoring and other classical decision-making methods. This comparison demonstrates that the fuzzy logic system outperformed traditional approaches in terms of accuracy and user satisfaction. By incorporating subjective preferences and variable data through fuzzy sets and rules, the system was better equipped to capture nuanced user needs than weighted scoring methods, which tend to assign fixed weights without accounting for inherent uncertainties or dynamic changes in user priorities (Table 6).

Table 6 demonstrates specific case evaluations comparing actual decisions to system recommendations:

- Case 1: The system’s recommendation perfectly matched the user’s choice (PLA), resulting in a 100% agreement;
- Case 2: While the system recommended PEEK, the actual decision was ABS, resulting in an 80% alignment based on overlapping criteria. This suggests that slight variations in user prioritization might benefit from more adaptive weight adjustments;
- Case 3 and Case 4: The system demonstrated a high degree of alignment (95% to 100%), underscoring the system’s effectiveness in aligning with user expectations.

The fuzzy logic system’s flexibility is particularly advantageous in handling subjective criteria, such as environmental impact and process scalability, where user preferences can vary significantly based on external market factors or specific production requirements. The following are some examples:

- Environmental Impact: The fuzzy system’s flexibility in managing sustainability-focused criteria allows SMEs to prioritize eco-friendly materials when desired. This adaptability is less feasible in fixed-weight traditional scoring methods.
- Process Scalability: By dynamically adjusting the importance of scalability, the fuzzy system provides tailored recommendations based on the production scale, which is critical for SMEs working across various project sizes.

The expert advisory system for 3D printing, currently in the testing phase, utilizes the AHP method to determine the weight of each assessment criterion. An expert analysis was conducted, and a preference matrix was created, which expresses the relative importance of each criterion in comparison to others. The matrix elements represent the preferences of each customer criterion (row) over a technical criterion (column) based on expert input.

The parameters for the selected 3D printing implementations were based on advisory input. For each task (such as selecting high strength or UV resistance), the best suitability for a given technology was determined.

In each case, different subsets of assessment criteria were used, depending on the input parameters. The domain of the criteria was calculated based on the identified parameters and used to form the support of the fuzzy sets. Using the AHP method, the weight of each active criterion was determined based on its relative preference.

The parameters and weights of the active criteria used in the decision-making process are summarized in Table 7. This table provides an overview of exemplary 3D printing campaigns, detailing the criterion type, weight, and the minimum and maximum values for each parameter.

The developed advisory system, integrating fuzzy logic and genetic algorithms, offers a structured solution for decision-making in the field of additive manufacturing, particularly for 3D printing. At its core, the system combines the AHP with fuzzy logic to handle imprecise data and subjective user preferences, ensuring flexible and precise support for complex industrial requirements. This multi-layered approach lays the groundwork for highly accurate recommendations aligned with user priorities and technical specifications [36].

The system’s operation begins with users defining key requirements related to additive manufacturing technologies. These requirements can range from technical specifications, such as UV resistance and thermal stability, to practical factors like material availability, production costs, and component precision. Each of these criteria is assigned a specific weight through the AHP method, resulting in a preference matrix that quantifies the relative importance of each factor based on expert input. This preference matrix, combined with calculated weights for active criteria, enables the system to generate well-calibrated recommendations for optimal 3D printing strategies tailored to user needs [37].

Once the criteria and their weights are established, the system uses fuzzy logic functions to model how well each potential technology option meets these criteria. To do so, it applies membership functions based on the MINSIMP and MAXSIMP criteria, depending on the nature of the requirement. For instance, in cases where high UV resistance is desirable, the system uses MAXSIMP to prioritize solutions with higher values. Conversely, for criteria like unit production cost, the system employs MINSIMP, which allows lower-cost solutions to be favored. Each criterion is transformed into a fuzzy set with specific membership functions that reflect the level of satisfaction each solution provides relative to the user’s requirements.

**Figure 2:**
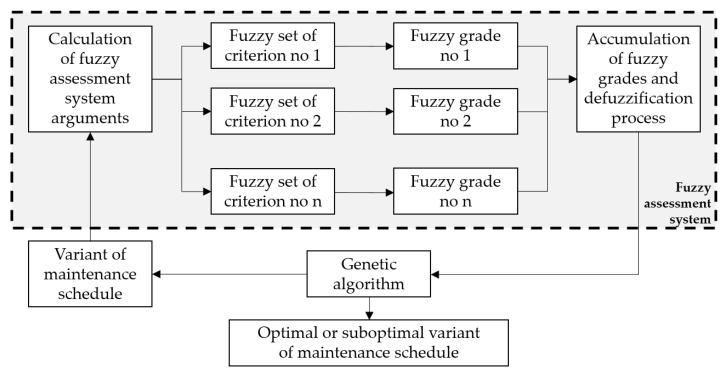
Scheme of a genetic-fuzzy expert system.

The previously described fuzzy assessment system was used to develop an automatic genetic-fuzzy system for the expert advisory system in technology selection. The structure of this system is presented in Figure 2.

This genetic-fuzzy system combines the principles of genetic algorithms with fuzzy logic to enhance the decision-making process. By integrating these two methodologies, the system can effectively optimize technology selection, accommodating the complexities and uncertainties inherent in the evaluation of various options. The structure depicted in Figure 2 highlights the key components and their interactions within the system, facilitating a clearer understanding of its functionality and application in real-world scenarios.

The analysis highlighted differing priorities: experts focused on the material’s availability and cost, which are crucial for short-term projects in SMEs, while the system considered long-term benefits such as durability and compliance with standards. Upon detailed review, it was concluded that the system’s recommendation was more appropriate in the context of long-term cost optimization and performance efficiency.

To verify the effectiveness of the fuzzy logic-based system, a statistical comparison was conducted against results from traditional weighted evaluation methods (Table 8). The analysis focused on two aspects:

- Recommendation accuracy (the percentage of matches between the system’s recommendations and actual user choices);
- User satisfaction (subjective user ratings on a scale from 1 to 10).

A *t*-test demonstrated that the differences between the methods were statistically significant (*p* < 0.05). To illustrate the system’s precision, confidence intervals at a 95% confidence level were added to membership values. The following are the key membership values for critical criteria:

- Material strength—average membership of 0.82 (95% CI: 0.78–0.86);
- Production cost—average membership of 0.75 (95% CI: 0.71–0.79).

The implementation of fuzzy logic in the advisory system demonstrates greater flexibility and adaptability to evolving user requirements compared to traditional weighted methods [38,39]. The findings suggest that fuzzy logic better accommodates subjective preferences and data uncertainty. Sensitivity analysis confirmed the system’s stability against minor weight changes in criteria, which is crucial for applications in dynamic production environments [40].

The case study underscores the importance of balancing the system’s intuitive usability with its ability to provide recommendations based on long-term benefits. Comparative results and confidence interval analyses emphasize the advantages of fuzzy logic-based systems, particularly for complex decisions that require evaluating numerous criteria.

## 4. Discussion

The conducted study highlights the significant advantages of using fuzzy logic to model the complex relationships between technical criteria and user preferences in the material selection process for additive manufacturing (AM). The developed advisory system demonstrates superior flexibility and accuracy compared to traditional decision-making methods, particularly in scenarios characterized by dynamically changing conditions and incomplete data. This adaptability is critical for the AM industry, which is marked by rapid technological advancements and the introduction of new materials.

However, despite its demonstrated effectiveness, the system has certain limitations. One notable challenge is its scalability when applied to a wide range of AM processes and materials. The system’s current structure relies on predefined membership functions and criteria weights, which may not fully capture the nuances of every AM process or material type. For instance, specific processes such as selective laser sintering or direct energy deposition require distinct technical considerations that differ from those for polymer-based AM technologies. Similarly, emerging materials like biocompatible polymers or advanced composites may necessitate updates to the system’s criteria and evaluation models. To address these issues, modular adaptations or process-specific configurations will need to be developed, ensuring the system remains applicable across diverse contexts.

Another limitation is the computational complexity that arises when scaling the system to handle a broader range of criteria or larger datasets. As the number of decision variables increases, the fuzzy inference process can become resource-intensive, potentially hindering real-time applications. This challenge could be mitigated by incorporating optimization techniques such as genetic algorithms or machine learning. These methods could dynamically refine the system’s parameters, enabling more efficient computation while maintaining decision accuracy [41].

The implementation of the advisory system in small- and medium-sized enterprises (SMEs) presents additional considerations. SMEs often operate with limited technical and financial resources, which can pose barriers to adopting advanced decision-support systems. While the system offers clear advantages, such as improving decision-making precision without requiring extensive expertise, its integration into SME workflows may require investment in compatible software, infrastructure, and training. Businesses with minimal digital capabilities or outdated systems could struggle with the initial adoption process. To overcome these barriers, the system must be designed with user-friendly interfaces and modular implementation options. Training programs tailored to SME needs and cloud-based platforms could further ease the integration process, reducing the dependency on local computational resources and making the system more accessible.

Despite these challenges, the study underscores the transformative potential of the fuzzy logic-based advisory system. By enabling more precise and informed material selection, even in the absence of detailed data, the system empowers SMEs to enhance their decision-making processes and compete effectively in the AM sector. Future advancements could further elevate its capabilities. For instance, integrating machine learning or genetic algorithms into the system could allow it to dynamically adapt to new user inputs or changes in market conditions, ensuring its continued relevance. Similarly, combining fuzzy logic with other decision-making frameworks, such as multi-criteria analysis or reinforcement learning, could result in hybrid systems that offer even greater precision and adaptability.

A case study was conducted to analyze situations where the advisory system’s recommendations diverged from expert opinions. One analyzed scenario involved selecting a material for thermally loaded components. Experts recommended using ABS, citing its widespread application and availability. In contrast, the system recommended PEEK, prioritizing criteria such as high thermal and chemical resistance [42].

## 5. Conclusions

The conducted research confirms the effectiveness of fuzzy logic as a robust decision-support tool within advisory expert systems for material selection in additive manufacturing, particularly addressing the unique needs of small- and medium-sized enterprises (SMEs). By facilitating nuanced decision-making under conditions of uncertainty and accommodating subjective user preferences, fuzzy logic proves invaluable in addressing the complexity of material selection—a process influenced by numerous technical, economic, and environmental factors. This approach provides SMEs with a more tailored and flexible means of decision-making, which is critical in an industry characterized by rapid advancements and the constant evolution of technology.

One of the key strengths of the system is its adaptability to user preferences, enabling decision-makers to prioritize criteria such as durability, cost, or environmental sustainability based on specific project needs. The fuzzy logic framework allows for the integration of subjective user inputs into the decision-making process, which is particularly valuable in SMEs where priorities can vary significantly between projects. This adaptability ensures that the system’s recommendations are not only technically sound but also aligned with the unique strategic goals and constraints of the user.

The broader implications for the AM industry are significant. In a field where rapid technological advancements and material innovations are the norm, the system’s ability to adapt to incomplete or imprecise data gives manufacturers a competitive edge. By supporting more accurate and flexible material selection, the system helps SMEs optimize production efficiency, reduce waste, and maintain high product quality. Furthermore, the integration of sustainability criteria aligns with the industry’s growing emphasis on environmentally responsible manufacturing, contributing to global efforts toward green technology and circular economy practices.

To maximize the system’s impact, its implementation in the additive manufacturing (AM) industry requires a carefully considered approach. Practical recommendations for deployment include customizing the system to meet sector-specific requirements, such as recyclability and scalability, and integrating it into existing enterprise resource planning (ERP) systems to streamline material selection workflows. Providing user training and ongoing support is essential to ensure that decision-makers can fully leverage the system’s functionalities. Additionally, pilot testing in real-world settings will allow for refinement and adaptation based on feedback, ensuring the system aligns closely with operational realities. Incorporating real-time data streams, such as material cost updates or performance metrics, will further enhance the system’s effectiveness by ensuring recommendations remain relevant and up-to-date.

Looking ahead, the future development of hybrid decision-support systems offers exciting possibilities for advancing material selection processes in AM. Incorporating machine learning (ML) algorithms into the system represents a significant opportunity to enhance its precision and adaptability. ML can analyze historical decisions and user feedback to refine criteria weights and membership functions dynamically, enabling the system to adapt to evolving user priorities and market demands. This adaptive capability would make the system more robust and responsive, ensuring its recommendations remain aligned with the latest trends and requirements.

Real-time data integration will also play a pivotal role in the system’s evolution. By connecting to external databases, IoT-enabled devices, and supplier networks, the system could access live data on material costs, availability, and performance, allowing SMEs to make well-informed, timely decisions. Expanding the system’s methodology to include additional multi-criteria decision-making (MCDM) techniques would further enhance its flexibility and utility in diverse decision-making scenarios.

To improve user experience, future iterations of the system should feature advanced visualization tools and intuitive interfaces that facilitate interactive decision-making. Visualization techniques, such as 3D modeling and dynamic graphs, would make the decision process more transparent, empowering users to better understand trade-offs between competing criteria. Reinforcement learning (RL) could be integrated to enable continuous improvement, allowing the system to refine its decision-making models based on real-time interactions and feedback.

Additionally, the development of domain-specific modules tailored to sectors like aerospace, healthcare, or automotive would enhance the system’s precision and applicability. Transitioning to a cloud-based platform would make the system more accessible and scalable, allowing SMEs to use it without significant infrastructure investments. Finally, incorporating sustainability metrics, such as carbon footprint and recyclability, would align the system with growing environmental priorities in the AM industry.

## Figures and Tables

**Table 1:** Interpretation of complete variant grades.

Complete Variant Grade	Interpretation
4	VL (Very Low)
5	L (Low)
6	ML (Medium Low)
7	M (Medium)
8	MH (Medium High)
9	H (High)
10	VH (Very High)

**Table 2:** Results of criterion weighting.

Criterion	Value Range	Weight
Material Strength	0.0–1.0	0.25
Production Cost	0.0–1.0	0.20
Environmental Impact	0.0–1.0	0.15
Durability	0.0–1.0	0.20
Process Scalability	0.0–1.0	0.20

**Table 3:** Correlation of customer criteria.

No	Criterion	Type
1	User safety	MINSIMP
2	Surface quality	MINSIMP
3	Dimensional accuracy	MINSIMP
4	Color range	MINSIMP
5	UV resistance	MINSIMP
6	Chemical resistance	MAXSIMP
7	Emission of toxic fumes	MINSIMP
8	Post-processing	MINSIMP
9	Abrasion resistance	MINSIMP
10	Biodegradability or recyclability	MINSIMP
11	Cost	MINSIMP
12	Market availability	MINSIMP
13	Compatibility with 3D printers	MAXSIMP
14	Ability to print at different speeds and settings without significant quality loss	MINSIMP
15	Thermal stability	MINSIMP
16	Compliance with standards and certifications	MAXSIMP

**Table 4:** Technical parameters.

No	Criterion	Type
a	Density [g/cm^3^]	MINSIMP
b	Glass transition temperature [°C]	MINSIMP
c	Coefficient of linear thermal expansion [1/°C]	MINSIMP
d	Moisture absorption [%]	MINSIMP
e	Processing shrinkage [%]	MINSIMP
f	Elongation at break [%]	MINSIMP
g	Elongation at yield [%]	MINSIMP
h	Elasticity [GPa]	MAXSIMP
i	Shore hardness [ShD]	MAXSIMP
j	Stiffness [GPa]	MAXSIMP
k	Tensile stress at break [MPa]	MAXSIMP
l	Yield stress [MPa]	MAXSIMP
m	Impact strength [J]	MAXSIMP
n	Young’s modulus [GPa]	MAXSIMP
o	Tensile strength [MPa]	MAXSIMP
p	Yield strength [MPa]	MAXSIMP

**Table 5:** The average degree of membership values assigned to each key criterion.

Criterion	Average Degree of Membership
Material Strength	0.82
Production Cost	0.75
Environmental Impact	0.80
Durability	0.70
Process Scalability	0.78

**Table 6:** Comparison of actual decisions with system recommendations.

Case	Actual Decision	System Recommendation	Agreement (%)
1	PLA (Czech Republic)	PLA	100
2	ABS (The Netherlands)	PEEK	80
3	PEEK (Poland)	PEEK	100
4	TPU (PA, USA)	ABS	95

**Table 7:** Parameters of active criteria for exemplary 3D printing campaign.

Criterion	Type	Weight	Min Value	Max Value
Density [g/cm^3^]	MINSIMP	1	1.00	7.95
Glass transition temperature [°C]	MINSIMP	1	55.0	2750.0
Coefficient of linear thermal expansion [1/°C]	MINSIMP	1	7.5 × 10^−7^	0.000125
Moisture absorption [%]	MINSIMP	1	0.05	15.0
Processing shrinkage [%]	MINSIMP	1	0.005	17.5
Elongation at break [%]	MINSIMP	1	0.05	250.0
Elongation at yield [%]	MINSIMP	1	0.05	72.0
Elasticity [GPa]	MAXSIMP	1	0.015	395.0
Shore hardness [ShD]	MAXSIMP	1	45.0	97.0

**Table 8:** Comparison results.

Method	Recommendation Accuracy (%)	Average User Satisfaction
Fuzzy Logic	85	8.7
Traditional weighted method	73	7.5

## Data Availability

The original contributions presented in the study are included in the article, further inquiries can be directed to the corresponding author.
